# Mortality in Dutch hospitals: Trends in time, place and cause of death after admission for myocardial infarction and stroke. An observational study

**DOI:** 10.1186/1472-6963-8-52

**Published:** 2008-03-04

**Authors:** Laurentius CJ Slobbe, Onyebuchi A Arah, Agnes de Bruin, Gert P Westert

**Affiliations:** 1 Department of Public Health and Healthcare, National Institute for Public Health and the Environment, Antonie van Leeuwenhoeklaan 9, PO Box 1, 3721 MA Bilthoven, The Netherlands; 2 Department of Health and Care, Statistics Netherlands, Prinses Beatrixlaan 428, 2273 XZ Voorburg, The Netherlands; 3 Faculty of Social and Behavioural sciences, Tilburg University, Warandelaan 2, PO Box 90153, 5000 LE Tilburg, The Netherlands; 4 Department of Social Medicine, Academic Medical Center, University of Amsterdam, Meibergdreef 9, PO Box 22700, 1100 DE Amsterdam, The Netherlands

## Abstract

**Background:**

Patterns in time, place and cause of death can have an important impact on calculated hospital mortality rates. Objective is to quantify these patterns following myocardial infarction and stroke admissions in Dutch hospitals during the period 1996–2003, and to compare trends in the commonly used 30-day in-hospital mortality rates with other types of mortality rates which use more extensive follow-up in time and place of death.

**Methods:**

Discharge data for all Dutch admissions for index conditions (1996–2003) were linked to the death certification registry. Then, mortality rates within the first 30, 90 and 365 days following admissions were analyzed for deaths occurring within and outside hospitals.

**Results:**

Most deaths within a year after admission occurred within 30 days (60–70%). No significant trends in this distribution of deaths over time were observed. Significant trends in the distribution over place of death were observed for both conditions. For myocardial infarction, the proportion of deaths after transfer to another hospital has doubled from 1996–2003. For stroke a significant rise of the proportion of deaths outside hospital was found. For MI the proportion of deaths attributed to a circulatory disease has significantly fallen ovtime. Seven types of hospital mortality indicators, different in scope and observation period, all show a drop of hospital mortality for both MI and stroke over the period 1996–2003. For stroke the observed absolute reduction in death rate increases for the first year after admission, for MI the observed drop in 365-day overall mortality almost equals the observed drop in 30-day in hospital mortality over 1996–2003.

**Conclusion:**

Changes in the timing, place and causes of death following admissions for myocardial infarction and stroke have important implications for the definitions of in-hospital and post-admission mortality rates as measures of hospital performance. Although necessary for understanding mortality patterns over time, including within mortality rates deaths which occur outside hospitals and after longer periods following index admissions remain debatable and may not reflect actual hospital performance but probably mirrors transfer, efficiency, and other health care policies.

## Background

Mortality after admission is seen as an important indicator of hospital performance, and forms part of several sets of quality indicators [1,2]. Some systems of measuring hospital performance even rely exclusively on post-admission mortality rates to rank hospital quality [3]. However, there are several pitfalls when it comes to calculating these indicators. The first question to consider is the influence of trends in place and time of death on hospital mortality statistics. An observed decline in hospital mortality after a myocardial infarction (MI) or stroke could indicate better care, but could also point to earlier discharge or to an increased transfer between hospitals, with death occurring after discharge or transfer. For Canada it has been shown that excluding transfer cases changes performance ranking for MI [4]. A related problem is the risk of administrative errors related to transfer. An American study revealed that double counting of patients in routine statistics occurred in 10–15% of all inter-hospital transfer cases, which significantly influenced both hospitalization and mortality rates [5]. It has been argued that the influence of transfer on hospital mortality statistics has grown in recent years, due to the shortening of length of stay [6]. An analysis of UK data has shown that the proportion of 30-day mortality falling within the initial admission has actually decreased over time[7].

A second question is how to attribute deaths after admission to the cause of death. Hospital mortality rates as a measure of quality are usually evaluated in terms of the direct cause of morbidity, but this need not be the true cause of death. Country statistics of deaths are in most cases based on national death records. These often take a different view of the cause of death by taking the patient history into account, before admission to the hospital. This opens up room for discrepancy and conflicting interpretations of death rates. Studies in Denmark [8] and the UK [9] have shown that a fairly large proportion of deaths after a hospitalization for MI or stroke are attributed to different causes in death records.

A third question associated with the use of mortality quality indicators is to what extent patients should be followed up after discharge, especially if hospital discharge registrations are not linked with each other or with a national death certificate register, as is the case in the Netherlands. Without such a link-up the administrative burden of follow-up after discharge is high, with large differences in the effort hospitals put into this follow-up. For example, the Dutch Health Care Inspectorate (IGZ) asks hospitals to collect data on 30-day mortality after admission for myocardial infarction. However, in the last publicized outcomes for this indicator, [10] about 40% of hospitals could not provide data on mortality after discharge. An in-depth analysis of five Dutch hospitals commissioned by the IGZ [11] revealed that there were also large differences in the way hospitals interpreted the necessary follow-up after discharge or transfer, with some settling for including transfers, others ignoring these, while others also included death outside their hospital.

The goal of this study is to assess the importance of these three questions for the computation of mortality indicators after discharge in the Netherlands for two conditions: myocardial infarction and stroke. The first question, the influence of trends in place and time of death on hospital mortality statistics, will be addressed by classifying death cases after an hospital admission for these conditions according to time and place of death. The second question, how to attribute deaths after admission to the cause of death will be addressed by comparing the cause of hospital admission with the cause of death on the death certificate. The third question, the extent to which patients should be followed up for the computation of mortality indicators, will be addressed by computing seven different mortality indicators which differ in the extension of the follow-up and the associated administrative burden.

## Methods

Records from the Dutch hospital discharge register (LMR) for the period 1995–2003 were linked to the population register by Statistics Netherlands. The hospital discharge register is maintained by Prismant Utrecht. This register contains discharge data for all Dutch general and academic hospitals, and contains information on patient-characteristics (date of birth, gender, place of residence) and episode characteristics (discharge diagnosis, date of admission and discharge). More than 87% of all hospital discharges in this register were successfully linked at the micro-level to the population register[12] The linkage techniques and the reliability and usability of this dataset for statistical research have been described elsewhere[13,14].

In addition this combined set was linked to the Dutch death certificate register, maintained by Statistics Netherlands. This was necessary to establish the time and the cause of death. This linkage was facilitated by the fact that both datasets use the same personal identifier, thus yielding almost 100% linkage rates after excluding those who emigrated abroad since admission to the hospital.

Approval for the use of the anonymized patient data was covered by a general agreement between Statistics Netherlands and Prismant. In addition, the Dutch association of hospitals (NVZ) approved the use of the hospital registration data for this study. No separate ethical approval was necessary for the use of these data.

This combined dataset was used to analyze the place, time, cause and rate of death within the first year after an index-admission for myocardial infarction or stroke among people aged 35 years and above. Index cases were defined using the main discharge diagnosis. The LMR uses the ICD9-CM (Dutch Clinical modification[15]) to register discharge diagnosis. Myocardial infarction was defined as ICD9-CM code 410, stroke as ICD9-CM code 431–434 and code 436.

Inclusion criteria for cases in the analysis were (a) having a principal diagnosis for myocardial infarction or stroke, (b) being 35 years or older at the end of the year of admission. Exclusion criteria were: (1) having another admission for the specified condition within 365 days before the index-admission; (2) having no hospital data available for any part of the period between 365 days before and 365 days after index admission; (3) ambiguously identified patients in the linked set in the year before and after the index-admissions, in order to avoid having administrative twins or émigrés; (4) if the date of mortality on the death certificate preceded the date of admission (as was noticed for 11 MI and 6 stroke patients). The second exclusion criterion mentioned above was necessary in order to verify the place of death (inside or outside the hospital) and to verify that an index-admission was not preceded within a year by a previous admission. This criterion implied that the first and last year of the dataset were used for verification purposes only, and did not yield any index-admissions. The third criterion led to the exclusion of 5% of previously selected cases.

Table 1 sums up some characteristics for selected index-cases.

**Table 1:** Characteristics of patients included in analysis & general characteristics Dutch hospitals & Dutch population

Admission characteristics*				
period	1996–2003			
diagnosis	Myocardial infarction		Stroke	
agegroup	[35–74]	[75+]	[35–74]	[75+]
				
Number of hospital admissions within LMR (before linking)	151,104	60,853	104,222	85,098
Number of hospital admissions within linked LMR	134,272	56,737	91,769	75,656
link yield (%)	88.9	93.2	88.1	88.9
				
Index cases selected before application mortality restriction	111,204	49,653	75,424	67,118
Index cases selected after application mortality restriction (death within a year of admission)	13,662	19,328	16,089	31,304
				
Characteristics index-cases				
Mean age	60.4	81.0	63.1	81.7
Proportion male (%)	74.5	49.9	60.7	41.8
Length of stay (days)	8.3	9.6	14.8	21.6
Decrease length of stay 1996–2003 (%)	-10.2	-7.2	-29.6	-30.4

**General characteristics Dutch hospitals**^**†**^				

	year			
	1996	2003		
Number of hospitals (general, academic, categorical)	148	129		
Number of beds (clinical & day care)	58,135	52,292		
Number of clinical admissions (thousands)	1,589	1,602		
Number of clinical hospital days (thousands)	15,531	12,757		
Number of day care admissions (thousands)^§^	705	1,221		
Workforce (full time equivalents, thousands)	139	175		

**General characteristics Dutch population**^**†**^				

	year			
	1996	2003		
average population size ages 35–74	7.1	7.9		
average population size ages 75+	0.9	1.0		
				
persons treated in a hospital for cva (ICD9 430–434, 436–438] per 10,000 population^‡^	15.1	15.4		
persons treated in a hospital for coronary heart disease [ICD9 410–414] per 10,000 population^‡^	45.4	39.8		

* source: this study

^† ^source: CBS [21]

^‡ ^standardized on Dutch population 1–1–2000.

^§ ^source: LMR. Prismant Utrecht

For all cases, the time to death was computed by subtracting the date of admission to a hospital from the date of mortality on the death certificate. All selected index-admissions were assigned a time of death class (within 0–29 days after admission, within 30–89 days after admission, within 90–365 days after admission), counting the date of admission as zero. For this analysis only those index cases resulting in death within a year of admission were used. We used chi-squared tests to detect significant correlations between year of admission and time of death and place of death categorizations.

All cases were also assigned a place of death class using any of four groupings:

-deaths within the index-admission

-deaths within a subsequent admission in the same hospital as the index-admission

-deaths that occurred in a different hospital

-deaths outside hospital

The cause of death was established using the primary cause registered on the death certificate, using the ICD-10 classification. Causes of death were grouped into three:

-cause of death attributed to cause of index-admission

-cause of death attributed to a circulation disorder other than index-condition

-deaths due to other causes

The difference in classification systems used in our morbidity data (ICD-9) and mortality data (ICD-10) caused a minor problem in establishing the correspondence between the cause of mortality and the index-condition for stroke because no exact translation could be made. We, therefore, decided to compare the outcomes with a slightly broader ICD-10 definition of stroke [I61–I69], also including indeterminate types. We used chi-square analysis to detect significant correlations between year of admission and cause of death.

In our data follow-up of patients was possible for a year after discharge. To show the effect of including or excluding different types of follow-up on mortality rates, we calculated seven different types of mortality rates, for each year in our dataset. Thirty-day mortality within the initial admission was easily calculated in addition to three period (30-, 90- and 365-day) rates were calculated for deaths in the hospital setting only (including transfers and readmissions) and for deaths outside the hospital. The denominator for each rate was the total number of admissions fulfilling index-conditions, including those still alive 365 days after the start of the initial admission. Rates were standardized for age and sex using the composition of the Dutch clinical hospital population in the year 2000.

## Results

Actual linkage rates of hospital discharge records to population register data were somewhat higher than average for the selected cases. Of all admissions in 1996–2003 for AMI age [35–74] 88.9% could be linked, for 75+ this was 93.2%. For stroke, linkage rates were somewhat lower, with 88.1% for 35–74 age category and 88.9% for those aged 75+ (Table 1).

For the period 1996–2003 we included 32,990 deaths after admission for MI and 47,393 deaths after admission for stroke in our analysis. Of the MI cases 67.9% of those aged 35–74 died within the first thirty days after admission, compared to total deaths within a year. For those aged 75+ this was 66.1%. For stroke age differences were larger with 67.6% dying within 30 days for ages 35–74 and 60.2% for ages 75+. These proportions are all stable over time: no significant differences between years were detected over the period 1996–2003.

Table 2 lists the breakdown of MI deaths in the different time of death and place of death classes and the year of admission, (given in two-year bands). Table 3 gives a similar breakdown for stroke. Chi-squared tests were used to detect significant trends over time, these are indicated within the tables.

**Table 2:** Dutch in-hospital mortality for myocardial infarction 1996–2003: deaths tabulated by age and place of death within 30, 90 and 365 days of admission

	**Deaths during initial admission**		**Deaths in same hospital. during subsequent admission**		**Deaths in a different hospital**		**Deaths outside hospital**		**All locations**	
**Time after admission**	**N**	**%**	**N**	**%**	**N**	**%**	**N**	**%**	**N**	**%**

**Ages 35–74**										
**deaths in 0–29 days**	*				*					

**1996–1997**	2,257	86.8	68	2.6	153	5.9	123	4.7	2,601	100.0
**1998–1999**	1,967	82.1	69	2.9	207	8.6	153	6.4	2,396	100.0
**2000–2001**	1,793	79.9	81	3.6	242	10.8	128	5.7	2,244	100.0
**2002–2003**	1,627	79.7	75	3.7	223	10.9	116	5.7	2,041	100.0

**deaths in 30–89 days**							^†^			

**1996–1997**	47	10.5	121	27.0	65	14.5	215	48.0	448	100.0
**1998–1999**	50	14.6	110	32.2	44	12.9	138	40.4	342	100.0
**2000–2001**	42	12.5	99	29.4	63	18.7	133	39.5	337	100.0
**2002–2003**	37	11.2	115	34.8	65	19.7	113	34.2	330	100.0

**deaths in 90–364 days**										

**1996–1997**	4	0.4	317	35.3	137	15.3	439	48.9	897	100.0
**1998–1999**	3	0.4	242	33.1	110	15.0	376	51.4	731	100.0
**2000–2001**	3	0.4	247	35.2	113	16.1	338	48.2	701	100.0
**2002–2003**	5	0.8	203	34.2	94	15.8	292	49.2	594	100.0

**Ages 75+**										
**deaths in 0–29 days**	*		^†^		*		^†^			

**1996–1997**	2,956	89.3	79	2.4	87	2.6	188	5.7	3,310	100.0
**1998–1999**	2,869	88.5	92	2.8	102	3.1	178	5.5	3,241	100.0
**2000–2001**	2,674	86.4	118	3.8	130	4.2	173	5.6	3,095	100.0
**2002–2003**	2,618	83.7	119	3.8	168	5.4	222	7.1	3,127	100.0

**deaths in 30–89 days**										

**1996–1997**	80	14.2	173	30.7	45	8.0	265	47.1	563	100.0
**1998–1999**	73	12.6	170	29.3	66	11.4	271	46.7	580	100.0
**2000–2001**	88	15.8	161	28.9	61	11.0	247	44.3	557	100.0
**2002–2003**	69	12.2	161	28.4	49	8.7	287	50.7	566	100.0

**deaths in 90–364 days**										

**1996–1997**	5	0.5	368	33.8	103	9.5	613	56.3	1,089	100.0
**1998–1999**	9	0.9	330	31.8	114	11.0	584	56.3	1,037	100.0
**2000–2001**	8	0.8	329	31.2	96	9.1	620	58.9	1,053	100.0
**2002–2003**	7	0.6	377	34.0	88	7.9	638	57.5	1,110	100.0

* 2-sided chi-square test trend significant p < .001.

^† ^2-sided chi-square test trend significant p < .05

**Table 3:** Dutch In-hospital mortality for stroke 1996–2003: deaths tabulated by age and place of death within 30, 90 and 365 days of admission

	**Deaths during initial admission**		**Deaths in same hospital, during subsequent admission**		**Deaths in a different hospital**		**Deaths outside hospital**		**All locations**	
**Time after admission**	**N**	**%**	**N**	**%**	**N**	**%**	**N**	**%**	**N**	**%**

**Ages 35–74**										
**deaths in 0–29 days**	^†^						^†^			

**1996–1997**	2,609	94.0	31	1.1	80	2.9	56	2.0	2,776	100.0
**1998–1999**	2,555	94.2	32	1.2	77	2.8	49	1.8	2,713	100.0
**2000–2001**	2,520	93.3	28	1.0	93	3.4	60	2.2	2,701	100.0
**2002–2003**	2,463	91.8	46	1.7	86	3.2	87	3.2	2,682	100.0

**deaths in 30–89 days**	*						*			

**1996–1997**	238	44.8	94	17.7	36	6.8	163	30.7	531	100.0
**1998–1999**	192	42.0	92	20.1	26	5.7	147	32.2	457	100.0
**2000–2001**	162	35.8	77	17.0	35	7.7	178	39.4	452	100.0
**2002–2003**	124	26.1	99	20.8	31	6.5	222	46.6	476	100.0

**deaths in 90–364 days**	*		^†^		^†^		^†^			

**1996–1997**	38	4.3	271	30.7	67	7.6	506	57.4	882	100.0
**1998–1999**	46	5.3	243	28.2	82	9.5	490	56.9	861	100.0
**2000–2001**	37	4.8	189	24.5	79	10.2	467	60.5	772	100.0
**2002–2003**	10	1.3	194	24.7	67	8.5	515	65.5	786	100.0

**Ages 75+**										
**deaths in 0–29 days**	*						*			

**1996–1997**	4,239	94.7	28	0.6	42	0.9	168	3.8	4,477	100.0
**1998–1999**	4,375	94.7	30	0.6	53	1.1	161	3.5	4,619	100.0
**2000–2001**	4,526	93.5	43	0.9	74	1.5	198	4.1	4,841	100.0
**2002–2003**	4,453	90.4	59	1.2	74	1.5	342	6.9	4,928	100.0

**deaths in 30–89 days**	*				^†^		*			

**1996–1997**	668	52.5	97	7.6	19	1.5	488	38.4	1,272	100.0
**1998–1999**	629	50.2	102	8.1	42	3.3	481	38.4	1,254	100.0
**2000–2001**	641	49.1	115	8.8	49	3.8	501	38.4	1,306	100.0
**2002–2003**	413	29.1	143	10.1	28	2.0	836	58.9	1,420	100.0

**deaths in 90–364 days**	*						*			

**1996–1997**	105	6.0	266	15.1	60	3.4	1,328	75.5	1,759	100.0
**1998–1999**	184	10.4	274	15.4	81	4.6	1,235	69.6	1,774	100.0
**2000–2001**	122	6.7	274	15.1	74	4.1	1,350	74.2	1,820	100.0
**2002–2003**	37	2.0	289	15.8	63	3.4	1,445	78.8	1,834	100.0

* 2-sided chi-square test trend significant p < .001.

† 2-sided chi-square test trend significant p < .05

For MI the analysis points to a growing importance of 'other hospitals' as death location for MI, especially for 30-day mortality. In 1996–1997 about 5.9% of the 30-day mortality after MI for ages 35–74 occurred in a hospital different from that of the initial intake, in 2002–2003 this proportion had significantly risen to 10.9%. This rise was at the expense of 30-day mortality within the initial admission, the proportion of which fell from 86.8% to 79.7% over the same period. For ages 75+ a similar trend is found, but somewhat less strong, although still significant.

No significant changes were detected for MI for other death locations or different distances between time of admission and time of death, with the exception of the proportion of deaths outside the hospital for ages 35–74 within 30–89 days after admission, this fell from 48.0% in 1996–1997 (215 deaths) to 34.2% in 2002–2003 (113 deaths).

For stroke a different picture emerges. No significant changes here for deaths in a different hospital, but a significant rise for deaths outside the hospital for both age groups and all three distance to death classes. The rise is especially strong for the proportion of deaths outside the hospital within 30–89 days of admission, and seems to be concentrated in the last years included in the analysis. For instance for deaths of 75+ within 30–89 days after being admitted the proportion of deaths outside the hospital was stable at 38.4% over 1996–2001, but rose steeply to 58.9% in 2002–2003. It is important to note that the observed 30% fall in average length-of-stay for stroke patients (Table 1) over the period 1996–2003 also occurred mainly in the last four years of this period.

Table 4 (MI) and Table 5 (stroke) list deaths for year of admission, time of death and cause of death. The patterns for both age groups are very similar, so data are presented for 35+. For admissions for MI this shows a significant (p < 0.05) decrease over time in the proportion of deaths attributed on the death certificate to MI, for all time of death classes. For deaths within 30-days of admission this decrease is accompanied by a significant rise in deaths due to other circulatory disorders and deaths due to other causes. For stroke only for deaths within 90–364 days of admission a similar pattern is found. For deaths due to stroke within 90 days of admission, no significant changes in the distribution of death cases are observed. For both MI and stroke, the diagnostic groups which contribute the most to 'other causes' are neoplasms, disorders of the endocrine system, and respiratory diseases.

**Table 4:** Underlying cause of death in people who died after hospital admission for myocardial infarction

	**Deaths due to AMI [I21-I22]**		**Deaths due to other circulatory disorders**		**Deaths due to other causes**		**all causes**	
**Time after admission**	**N**	**%**	**N**	**%**	**N**	**%**	**N**	**%**

**Ages 35+**								
**deaths in 0–29 days**	*		*		*			

**1996–1997**	4,533	76.7	834	14.1	544	9.2	5,911	100.0
**1998–1999**	4,219	74.8	863	15.3	555	9.8	5,637	100.0
**2000–2001**	3,839	71.9	865	16.2	635	11.9	5,339	100.0
**2002–2003**	3,632	70.3	900	17.4	636	12.3	5,168	100.0

**deaths in 30–89 days**	*				^†^			

**1996–1997**	392	38.8	385	38.1	234	23.1	1,011	100.0
**1998–1999**	323	35.0	384	41.6	215	23.3	922	100.0
**2000–2001**	310	34.7	350	39.1	234	26.2	894	100.0
**2002–2003**	263	29.4	377	42.1	256	28.6	896	100.0

**deaths in 90–364 days**	*				*			

**1996–1997**	541	27.2	804	40.5	641	32.3	1,986	100.0
**1998–1999**	444	25.1	743	42.0	581	32.9	1,768	100.0
**2000–2001**	394	22.5	697	39.7	663	37.8	1,754	100.0
**2002–2003**	326	19.1	720	42.3	658	38.6	1,704	100.0

* 2-sided chi-square test trend significant p < .001.

† 2-sided chi-square test trend significant p < .05

**Table 5:** Underlying cause of death in people who died after hospital admission for stroke

	**Deaths due to cva [I61-I69] excl. subarachnoid hemorrhage**		**Deaths due to other circulatory disorders**		**Deaths due to other causes**		**all causes**	
**Time after admission**	**N**	**%**	**N**	**%**	**N**	**%**	**N**	**%**

**Ages 35+**								

**deaths in 0–29 days**								

**1996–1997**	5,395	74.4	898	12.4	960	13.2	7,253	100.0
**1998–1999**	5,459	74.5	823	11.2	1,050	14.3	7,332	100.0
**2000–2001**	5,651	74.9	838	11.1	1,053	14.0	7,542	100.0
**2002–2003**	5,717	75.1	878	11.5	1,015	13.3	7,610	100.0

**deaths in 30–89 days**	^†^							

**1996–1997**	1,070	59.3	270	15.0	463	25.7	1,803	100.0
**1998–1999**	989	57.8	265	15.5	457	26.7	1,711	100.0
**2000–2001**	1,012	57.6	281	16.0	465	26.5	1,758	100.0
**2002–2003**	1,036	54.6	311	16.4	549	29.0	1,896	100.0

**deaths in 90–364 days**	^†^				^†^			

**1996–1997**	1,044	39.5	563	21.3	1,034	39.2	2,641	100.0
**1998–1999**	1,064	40.4	513	19.5	1,058	40.2	2,635	100.0
**2000–2001**	1,024	39.5	514	19.8	1,054	40.7	2,592	100.0
**2002–2003**	931	35.5	529	20.2	1,160	44.3	2,620	100.0

* 2-sided chi-square test trend significant p < .001.

† 2-sided chi-square test trend significant p < .05

In Table 6 and Table 7, mortality rates are presented for both types of index-admissions and both age-groups. Rates were standardized using the average age and sex composition of the clinical hospital population in 2000. In addition, we estimated mortality rate changes (as absolute differences between rates) between 1996 and 2003. Most important observation is that all mortality rates have fallen over this period, but the magnitude of this fall differs. For MI, the highest reduction is observed for 30-day in-hospital mortality. After including readmission and transfer cases, this decrease is much less. For instance, hospital mortality after MI for ages 35–74 has fallen from 7.1 to 5.8 percent, over 1996–2003, a drop of 1.3%, including other 30-day hospital deaths reduces this to 0.9%. Overall 365-day mortality dropped by 1.2%, a larger amount than both 30-day overall mortality (0.8%) and 90-day overall mortality (0.8%). For ages 75+ the picture for MI is the same, but much higher absolute gains in mortality reduction are found at higher levels of mortality. The 30-day in-hospital mortality for 75+ has dropped from 24.2% to 20.2%, a drop of 4.1%. Including other 30-day hospital deaths reduces this drop to 3.1%. Again, 365-day overall mortality dropped by 3.7% further than 30-day overall mortality (3.1%) and 90-day mortality (3.4%). Observed drops in MI-mortality rates occur in most cases gradually over the entire observation period.

**Table 6:** Mortality rates* 1996–2003 after admission for myocardial infarction, for seven different definitions of mortality, as percentage of admissions

	**30- day mortality initial admission**	**30- day mortality initial + subsequent admissions**	**30-day mortality all locations of death**	**90- day mortality initial + subsequent admissions**	**90-day mortality all locations of death**	**365- day mortality initial + subsequent admissions**	**365-day mortality all locations of death**
**a) mortality rates ages 35–74**							

**1996–1997**	7.1	7.7	8.1	8.3	9.3	9.5	11.5
**1998–1999**	6.6	7.6	8.0	8.3	9.1	9.4	11.2
**2000–2001**	6.5	7.6	8.0	8.3	9.0	9.4	11.2
**2002–2003**	5.8	6.9	7.3	7.6	8.3	8.6	10.3
							
**difference 2003–1996**	-1.3	-0.9	-0.8	-0.8	-0.9	-0.9	-1.2

**b) mortality rates ages 75+**							

**1996–1997**	24.2	25.6	27.1	28.1	31.8	31.8	40.5
**1998–1999**	23.3	24.9	26.3	27.4	31.0	31.0	39.2
**2000–2001**	21.7	23.7	25.1	26.3	29.6	29.7	38.1
**2002–2003**	20.2	22.4	24.1	24.5	28.4	28.1	36.8
							
**difference 2003–1996**	-4.1	-3.2	-3.1	-3.6	-3.4	-3.8	-3.7

*rates standardized for average Dutch hospital population 2000

**Table 7:** Mortality rates* 1996–2003 after admission for stroke, for seven different definitions of mortality, as percentage of admissions

	**30- day mortality initial admission**	**30- day mortality initial + subsequent admissions**	**30-day mortality all locations of death**	**90- day mortality initial + subsequent admissions**	**90-day mortality all locations of death**	**365- day mortality initial + subsequent admissions**	**365-day mortality all locations of death**
**a) mortality rates ages 35–74**							

**1996–1997**	12.3	13.0	13.3	14.5	15.4	16.1	18.8
**1998–1999**	12.5	13.2	13.4	14.4	15.3	15.9	18.7
**2000–2001**	12.5	13.2	13.4	14.2	15.2	15.5	18.4
**2002–2003**	11.4	12.1	12.4	13.1	14.3	14.2	17.3
							
**difference 2003–1996**	-0.9	-0.9	-0.9	-1.4	-1.1	-1.9	-1.5

**b) mortality rates ages 75+**							

**1996–1997**	26.5	26.9	27.9	31.8	35.8	34.5	46.8
**1998–1999**	26.5	27.0	27.9	31.7	35.5	35.0	46.3
**2000–2001**	26.3	26.9	28.0	31.6	35.5	34.4	46.1
**2002–2003**	23.5	24.2	26.0	27.3	33.4	29.4	43.0
							
**difference 2003–1996**	-3.0	-2.7	-2.0	-4.5	-2.5	-5.1	-3.8

*rates standardized for average Dutch hospital population 2000

For stroke a slightly different picture emerges. Reduction of 30-day mortality within the initial admission is lower than the observed drop for 365 day mortality. For ages 35–74, 30-day mortality within the initial admission has fallen from 12.3 to 11.4%, a drop of 0.9%, while 365-day overall mortality has fallen with 1.5%. For ages 75+, 30-day mortality within the initial admission has fallen from 26.5 to 23.5%, a drop of 3.0%, while 365-day overall mortality has fallen with 3.8%. For stroke, the observed reduction occurs in the last two years of the observation period, but not before.

## Discussion

In our introduction we identified three problems connected to the calculation of mortality indicators: influence of trends in place and time of death on mortality statistics, discrepancy between cause of admission and cause of death and administrative difficulties with the follow-up of patients after discharge.

Some limitations of the study need to be addressed. About 10% of MI and stroke records could not be linked to population and death registers, because people had administrative twins. However, research by Statistics Netherlands indicates that the influence of this on outcomes is limited [12]. Chances of having an administrative twin are somewhat higher in densely populated areas, and also differ by age. Another limit is of course that available care options are strongly influenced by government regulations, in the Dutch situation for instance the severe restrictions on the performance of cardiovascular procedures. However we do think our results may be of wider applicability. For instance our outcomes for place, time and cause of death analysis are for key elements, like the proportion of deaths occurring within the initial admission or the proportion of 30-day mortality compared to 1 year mortality, very similar to results of a UK study using data from the Oxford Record Linkage Study [9]. This is remarkable because of the large difference between the tax-funded health care system in the UK and the insurance based system in the Netherlands.

Regarding the question of incorporating transfer cases, we showed that in the Netherlands the proportion of deaths occurring after a transfer has grown for MI, especially for 30-day mortality. The proportion of deaths occurring in another hospital has almost doubled for this group between 1996 and 2003. However, for stroke transfer seems not to be an important issue.

Although it is likely the most severe cases with a high risk of mortality are transferred, it is important to stress that the observed increase over time in post-transfer deaths for MI does not necessarily imply an increase over time in mortality rates for transferred patients. Transfer between hospitals is not a random process but is driven by differences in the availability of resources like IC-units, or a difference in treatment options between hospitals. For instance, in the Netherlands only a minority of hospitals (~20%) are allowed to perform percutaneous translumminal coronary angioplasty (PTCA) after a myocardial infarction. Two competing explanations are possible here: mortality rates for cases after transfer are higher because the time between admission and treatment will be longer in general. But mortality rates could also be lower due to better treatment options available in the receiving hospital. Current research like the Prague-1 study [16,17] suggests that the beneficial effects of transfer outweigh negative effects.

The second problem, discrepancy between the cause of hospital admission and the cause of death seems to be an issue growing in importance for deaths after MI but not for stroke. Over the observation period the number of deaths after an MI attributed to another circulatory disease but especially to 'other causes' has grown significantly. The reason for this is unknown. Part of the explanation could be that the accuracy of the death certificates issued after a MI have improved with more cases in which MI can be seen as a complication rather than a primary cause being attributed to the 'true' cause. Another possibility could be a change in diagnostic procedures or in the case-mix patients being admitted. Over the period 1996–2003, the age-standardized rate of persons treated in hospital for coronary heart disease fell from 45.4 per 10,000 population to 39.8 per 10,000 (Table 1). No such decline is observed for stroke. It could be the case that improved prevention has especially benefited people with a coronary heart disease.

As for the third area, follow-up difficulties, this analysis demonstrates that it is possible to calculate a wide range of hospital mortality indicators using linked morbidity and mortality data.

Using longer time-frames and adding deaths beyond the initial admission increases the number of mortality cases found for both MI and stroke. However, our results show that there is no substantial difference in trends between easily computed 30-day in-hospital mortality, and other indicators which require more effort to compute. All the indicators point to a decline in hospital mortality for MI and stroke for the observed period, with most of the deaths occurring within 30 days of the first admission. For MI it is advisable to exclude transferred cases from the computation of mortality indicators, because of the significant rise of deaths occurring after transport observed over the study period. This is also in line with emerging literature where exclusion of transfer cases does not appear to alter the main conclusions about hospital performance (Peterson et al [18], Bradley et al (19)). For the specific evaluation for the effect of transfer on mortality rates of transferred patients – very likely more severe cases – a separate indicator could be constructed.

Nonetheless, useful insights can be gathered from computing other indicators which cover other places of death beyond index hospitals and longer time frames. Over the observed period, death rates have dropped. It is interesting to observe that the absolute gain improvement in 30-day in-hospital mortality from the period 1996 to 2003 is comparable to the absolute improvement in 365-day mortality (all locations) for MI. This suggests that most of the improvement in survival is reached within the initial hospital treatment period, and that this is a long term effect because no substantial decrease in survival is observed after these first 30 days, which would be the case of there was only a short-term effect of better initial treatment. For stroke the markedly better improved survival rate after one year (as compared to 30-day mortality) indicates that for this condition improvement of care after discharge from the hospital plays a substantial role in the better survival. The almost universal implementation in the last years of our observation period of 'stroke units' in the Netherlands, aimed at optimizing the integration of pre- and post discharge care for stroke, seems to be very successful. For both MI or stroke, there is room for substantial improvement due to secondary prevention after the initial event.

This sheds light on a debate in the Dutch research community [19] about the cause of the declining mortality in the Netherlands for myocardial infarction: better primary prevention of hearth disease or better post-myocardial infarction treatment with increased use of angioplasties and drugs like statins.

Since 30-day in-hospital mortality correlates well with other types of mortality, it underlines the continuing importance of this indicator as a measure of hospital performance in terms of MI and stroke treatments they provide.

## Conclusion

More research is needed to link actual hospital processes to these outcome measures, considering the mixed results recently reported in the literature [18,20]. Therefore, our study cautions against expecting too much from the use of 30-day in-hospital mortality as an indicator, as our research shows it is well worth to study hospital mortality in the broader context of total mortality, and follow-up mortality over longer time-frames. Although necessary for understanding mortality patterns over time, including within mortality rates deaths which occur outside hospitals and after longer periods following index admissions remain debatable and may not reflect actual hospital performance but probably mirrors transfer, efficiency, and other health care policies.

## Competing interests

The author(s) declare that they have no competing interests.

## Authors' contributions

LCJS performed the analysis, interpreted the results, and drafted the manuscript. OAA designed the study, helped interpret the results, and contributed to the discussion. AB provided and supervised preparation of data used in the study. GPW supervised the study and participated in the formulation of the discussion. All authors reviewed and edited the manuscript for intellectual content.

## Pre-publication history

The pre-publication history for this paper can be accessed here:


